# Community–Based Epidemic Prevention in Taiwan: Combating the Coronavirus Disease–2019 Crisis

**DOI:** 10.1017/dmp.2020.146

**Published:** 2020-05-07

**Authors:** Huei-Wen Angela Lo, Joh-Jong Huang, Cheng-Chung Chen, Duujian Tsai, Frank Huang-Chih Chou, Vincent Shieh

**Affiliations:** Faculty of Medicine, College of Medicine, Kaohsiung Medical University, Kaohsiung, Taiwan; Department of Family Medicine, Kaohsiung Medical University, Chung-Ho Memorial Hospital, Kaohsiung, Taiwan; Kaohsiung Municipal Kai-Syuan Psychiatric Hospital, Kaohsiung, Taiwan; Pingtung Christian Hospital, Pingtung, Taiwan; Institute of Health and Welfare Policy, National Yang Ming University, Taipei, Taiwan; Graduate Institute of Gender Education, National Kaohsiung Normal University, Kaohsiung, Taiwan

**Keywords:** community-based epidemic prevention, COVID-19, Taiwan

Taiwan is on the Pacific Ring of Fire where frequent earthquakes and seasonal monsoons contribute to frequent natural disasters, and in a destruction-reconstruction-destruction continual cycle. In the past decades, Taiwan has experienced many natural, human created, biological, and compound disasters. In order to help prevent as well as manage these disasters, the Executive Yuan (Taiwan’s executive governing authority)^1^ has developed a Disaster Management White Paper; this paper provides strategies for disaster management with an emphasis on raising community awareness, establishing disaster prevention procedures, and building disaster-resistant and sustainable communities.

As the number of coronavirus disease–2019 (COVID-19) cases expanded and the death toll dramatically increased, the World Health Organization^2^ announced the global epidemic as a “public health emergency of international concern.” The policies of quarantine, social distancing, and area lockdowns were adapted by many countries. Taiwan is also in a state of emergency and working to mobilize community resources and identify solutions. Taiwan is using community self-monitoring as a foundational strategy and practicing other epidemic prevention strategies to reduce the risks of spreading the COVID-19 virus. We are implementing a “local hospital-community” mutual support system.

Based on Taiwan’s past experiences with epidemic prevention, the following recommendations were developed regarding community risk evaluation and management:

1. Establish partnerships among community residents, health care experts, and epidemic-prevention professionals
2. Organize and develop a shared cloud-based platform that provides up to date and accurate information on the epidemic, disease prevention, and community medical care
3. Organize and implement suitable spaces for self-quarantine and isolation, as well as a community mutual-aid system
4. Establish public community systems for health enhancement, medical care, and epidemic prevention
5. Evaluate communities’ needs in epidemic prevention as a reference for planning and implementing disaster prevention and relief policies
6. Use smart technology to assist communities in monitoring citizens’ health and to maintain and improve the quality of life in communities
7. Provide financial support for supplies, psychological support, and ongoing needs assessment to reduce the community’s social vulnerability
8. Implement a comprehensive health professional task-shift project bridging hospital-clinical- community with sufficient technological support for community empowerment

During the COVID-19 pandemic, Taiwan has worked to strengthen community partnerships that support prevention and continually adapt as the circumstances of the outbreak change; another goal is to develop community capacity by promoting individual citizen self-monitoring. To address the impacts of biological disasters on communities, we adhere to the concept of “thinking globally, acting locally.” We actively participate in international epidemic-prevention communities to share Taiwan’s experiences and research regarding community epidemic-prevention procedures and risk-management models.
